# The Emerging Role of TYRO3 as a Therapeutic Target in Cancer

**DOI:** 10.3390/cancers10120474

**Published:** 2018-11-29

**Authors:** Sherri K. Smart, Eleana Vasileiadi, Xiaodong Wang, Deborah DeRyckere, Douglas K. Graham

**Affiliations:** 1 Department of Pediatrics, Emory University, Atlanta, GA 30322, USA; sherri.smart@choa.org (S.K.S.); eleana.vasileiadi@emory.edu (E.V.); deborah.deryckere@emory.edu (D.D.); 2 Aflac Cancer and Blood Disorders Center, Children’s Healthcare of Atlanta, Atlanta, GA 30322, USA; 3 Center for Integrative Chemical Biology and Drug Discovery, Division of Chemical Biology and Medicinal Chemistry, Eshelman School of Pharmacy, University of North Carolina, Chapel Hill, NC 27599, USA; xiaodonw@email.unc.edu

**Keywords:** TYRO3, MERTK, AXL, TAM family, receptor tyrosine kinase, targeted therapy, signaling pathway, cancer

## Abstract

The TAM family (TYRO3, AXL, MERTK) tyrosine kinases play roles in diverse biological processes including immune regulation, clearance of apoptotic cells, platelet aggregation, and cell proliferation, survival, and migration. While AXL and MERTK have been extensively studied, less is known about TYRO3. Recent studies revealed roles for TYRO3 in cancer and suggest TYRO3 as a therapeutic target in this context. TYRO3 is overexpressed in many types of cancer and functions to promote tumor cell survival and/or proliferation, metastasis, and resistance to chemotherapy. In addition, higher levels of TYRO3 expression have been associated with decreased overall survival in patients with colorectal, hepatocellular, and breast cancers. Here we review the physiological roles for TYRO3 and its expression and functions in cancer cells and the tumor microenvironment, with emphasis on the signaling pathways that are regulated downstream of TYRO3 and emerging roles for TYRO3 in the immune system. Translational agents that target TYRO3 are also described.

## 1. Introduction

TYRO3 is a member of the TAM (TYRO3, AXL, MERTK) family of transmembrane receptor tyrosine kinases [1], which share overlapping functions in tumorigenesis and suppression of anti-tumor immunity [2]. While roles for MERTK and AXL in these processes have been well-described, less is known about the roles for TYRO3. In addition, many studies have assessed the effects of TYRO3 inhibition in conjunction with inhibition of MERTK and/or AXL in an effort to evaluate overlapping functions and have not evaluated contributions of the individual family members. Similarly, studies describing the effects of TAM kinase ligands in biologic systems do not always evaluate the contributions of individual family members. Here we provide a brief review of what is known about specific roles for TYRO3 in normal physiology and a more comprehensive review of the current literature demonstrating roles for TYRO3 in tumorigenesis.

## 2. Structure, Expression, and Mutations

### 2.1. TYRO3 Structure

**Figure 1:**
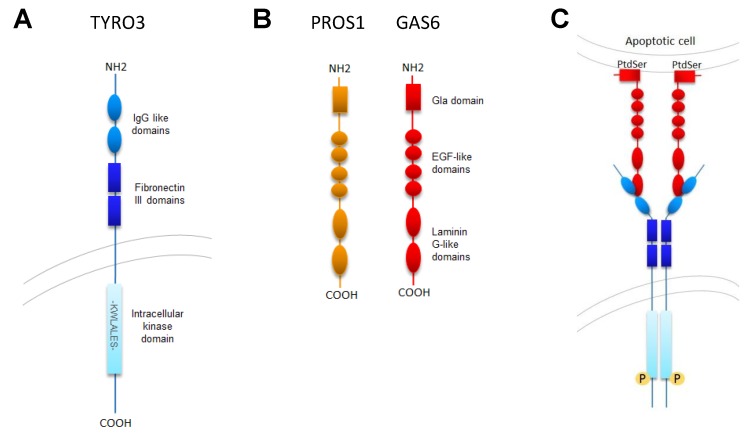
TYRO3 Structure and Activation. (**A**) TYRO3 is a 890 amino acid transmembrane protein composed of two extracellular IgG like domains (amino acids 60–117 and 156–203), two extracellular fibronectin III domains (amino acids 224–313 and 322–409), a transmembrane portion (amino acids 430–450) and an intracellular kinase domain (amino acids 525–776). The conserved KW(I/L)A(I/L)ES sequence in the kinase domain is unique to the TAM-family receptor tyrosine kinases. (**B**) The best characterized TYRO3 ligands are Protein S (PROS1) and GAS6, which share ~43% sequence homology and contain a Gla domain, 4 EGF-like domains, and two Laminin G-like domains. (**C**) Activation of TYRO3. PROS1 or GAS6 binds to phosphatidylserine on membranes of apoptotic and virus-infected cells and promotes dimerization, autophosphorylation and activation of TYRO3. Ligand independent activation of TYRO3 has also been reported.

TAM kinases have a distinct structure composed of an extracellular domain, a single helix transmembrane domain, and an intracellular domain (Figure 1A) [3,4,5,6]. The extracellular domain contains two fibronectin type III and two Ig domains and the cytoplasmic domain mediates kinase activity and acts as a binding site for other molecules. The family is further distinguished from other tyrosine kinases by the presence of a conserved KW(I/L)A(I/L)ES sequence in the kinase domain. Amino acids 3 and 5 within this sequence are leucine (L) in TYRO3 and isoleucine (I) in MERTK and AXL. TYRO3 was independently cloned by numerous groups and as a result, has been called by many names, including *TYRO3*, *SKY*, *RSE*, *BYK* and *TIF* in humans, *Dtk*, *Rse*, *Brt*, *Etk2* and *Tyro3* in mice, and *REK* in chickens [3,7]. The human TYRO3 protein contains 890 amino acids and has a predicted molecular weight of 97 kDa, although the actual molecular weight ranges between 120–140 kDa due to post-translational modifications, including glycosylation [8,9,10,11]. Two splice variants of TYRO3 have been demonstrated and a third predicted containing either exon 2A, 2B, or 2C, which encode the signal sequence in the extracellular domain and may be important for post-translational modifications, localization and function [6,8,12]. Next generation computational modeling methods that rely on publicly-available cDNA and mRNA data predicted the existence of up to 9 TYRO3 splice variants containing overlapping exons, alternative splicing or intron retention, although these have not been verified in cell-based assays [13].

### 2.2. Physiologic TYRO3 Expression

TYRO3 is physiologically expressed in a variety of tissues and is most prominent in the nervous system. In the brain, TYRO3 is found in endothelial cells [14], neurons [15,16,17], oligodendrocytes [18], and the hippocampus [19] and TYRO3 is also expressed in Schwann cells in the peripheral nervous system [20]. In the hematopoietic system, TYRO3 is expressed in dendritic cells [21,22], natural killer cells [23], monocytes and macrophages [22,24], platelets and megakaryocytes [25,26,27,28] and osteoclasts [29,30]. In the reproductive system, TYRO3 is expressed in male primordial germ cells and supporting cells in the gonads, Sertoli cells in the testes and granulosa cells in the ovaries [31]. TYRO3 is also expressed in the kidney, lung, skeletal muscle, liver, pancreas and myocardium [3,32]. The extracellular domain of TYRO3 can be shed from the cell membrane by proteolytic cleavage; however little or no soluble TYRO3 is detected in serum in the absence of disease states [33,34].

### 2.3. TYRO3 Expression in Cancer

Expression of TYRO3 has been noted in a variety of malignancies, including colon cancer [35,36], breast cancer [37,38,39,40], lung cancer [41,42,43,44], liver cancer [33,45,46], thyroid cancer [47], melanoma [48,49,50,51,52,53], schwannoma [54,55], ovarian cancer [56,57,58], prostate cancer [59,60], leiomyosarcoma [61,62], dedifferentiated liposarcoma [61], undifferentiated pleomorphic sarcoma [61], synovial sarcoma [61], esophageal cancer [63], endometrial cancer [64], multiple myeloma [65] and several leukemia subtypes [66,67,68]. In many cases TYRO3 was overexpressed relative to corresponding normal tissues. For example, TYRO3 mRNA levels were increased by 2-fold or greater in approximately 40% of hepatocellular carcinoma patient samples compared to adjacent normal liver tissue and in a panel of human liver cancer cell lines relative to a normal hepatocyte cell line [45]. Corresponding increases in protein levels were also observed. Similarly, TYRO3 protein levels were dramatically increased in primary human schwannoma cells relative to normal Schwann cells [55]. Abundant TYRO3 expression was also demonstrated in murine breast cancer tissue, while TYRO3 expression was minimal in normal mouse breast tissue [39]. TYRO3 and AXL were both expressed in a subset of cutaneous melanoma cell lines and in this context, expression of the two receptors was mutually exclusive [53]. TYRO3 was absent in normal thyroid tissue, but was ectopically expressed in all human thyroid carcinoma cell lines tested [47]. In hematologic malignancies, approximately half of acute myeloid leukemia (AML) patient samples expressed TYRO3 mRNA [66]. TYRO3 was also expressed in B and T-cell acute lymphoblastic leukemia (ALL) cell lines [66,67] and chronic lymphocytic leukemia patient samples [69]. Initial data suggest that TYRO3 expression is ectopic in ALL as normal B and T cells [22] and cell lines [66] do not appear to express TYRO3; however, a thorough analysis of TAM kinase expression in B and T cells has not been reported and it is not clear whether TYRO3 is expressed in specific phenotypic subsets or during specific phases of the immune response. TYRO3 was also expressed in liver metastases in patients with colorectal cancer [36] and in metastatic bone lesions in mice with a prostate cancer xenograft [60]. TYRO3 levels were elevated in serum from patients with prostate cancer or hepatocellular carcinoma, implicating TYRO3 as a potential biomarker [33,59]. A complete description of the literature describing TYRO3 expression in cancer is included in Table 1.

### 2.4. Upstream Regulators of TYRO3 Expression

Numerous upstream regulators of TYRO3 expression have been identified. TYRO3 mRNA and protein levels are increased in PC12 pheochromocytoma cells following induction of a neuronal phenotype in response to treatment with nerve growth factor (NGF) and increased expression is mediated via activation of the high affinity NGF receptor tropomyosin-related kinase A (TRKA) and downstream signaling through phosphatidylinositol-3-kinase (PI3K) [70]. Interestingly, TRKA and TYRO3 colocalized in PC12 cells after NGF treatment and ectopically-expressed TRKA and TYRO3 co-immunoprecipitated, suggesting a physical interaction and the possibility of an additional post-translational mechanism to increase TYRO3 expression in this context. Similarly, treatment with stromal cell derived factor 1α (SDF-1α), which is a ligand for the chemokine receptor CXCR4 [71], upregulated TYRO3 mRNA and protein expression in thyroid cancer cells and overexpression of moesin-ezrin-radixin-like protein (MERLIN), a membrane and cytoskeletal scaffolding tumor suppressor, in primary schwannoma cells led to decreased TYRO3 protein levels. While these upstream signaling proteins likely regulate TYRO3 mRNA levels indirectly, the microRNA miR-7-5p (miR-7) has been identified as a direct regulator of *TYRO3* gene expression [46,72]. Overexpression of miR-7 in hepatocellular and colorectal carcinoma cells significantly decreased TYRO3 mRNA and protein levels, and miR-7 seed sequences in the 3′ untranslated region of the *TYRO3* gene were essential for miR-7 regulation of luciferase from a TYRO3 reporter construct [46,72].

### 2.5. TYRO3 Mutations in Cancer

TYRO3 mutations have been noted in human malignancies, but functional studies have not been performed to understand the potential significance of these mutations. Mutations within the kinase domain have been identified in colon cancer (M592I) [73], lung cancer (N615K) [74], melanoma (W708fs*5) [75], brain cancer (A709T) [76], and AML (C690R) [77]. Cytosolic domain mutations have been found in melanoma (R462Q) [75], pancreatic cancer (R514Q) [78], and colon cancer (G809D) [73]. Mutations in the extracellular and transmembrane domains leading to a premature stop codon have been described in melanoma (Q67 and H60Q) [50,79] and lung cancer (E340) [74]. Missense or truncation mutations in *TYRO3* were also identified in 3 of 23 samples from patients with CML who developed resistance to BCR-ABL inhibitors while on therapy and did not have a resistance conferring mutation in *BCR-ABL* itself, although the significance of this finding is also unknown [80].

## 3. Activation and Cell Signaling

### 3.1. TYRO3 Ligands

TYRO3 is activated by two structurally-related ligands, growth arrest specific gene 6 (GAS6) and the anti-coagulation factor Protein S (PROS1) (Figure 1B) [81,82]. Both proteins require vitamin K dependent gamma-carboxylation to become biologically active and bind via their C-terminal domains to the ectodomain of TYRO3 to promote dimerization and induce TYRO3 auto-phosphorylation by/of the intracellular kinase domain [33,81,82]. The negatively charged gamma-carboxylated domain (Gla) of PROS1 and GAS6 forms complexes with 7–8 calcium ions, leading to exposure of phospholipid binding sites [83] and allowing formation of lipid-protein complexes consisting of GAS6 or PROS1 bound to phosphatidylserine (PS) on the surface of apoptotic cells, aggregating platelets, or enveloped viruses (Figure 1C) [84]. In studies using chimeric receptor-based reporter systems both PROS1 and GAS6 bound to TYRO3 in the absence of PS, but their affinity for TYRO3 was significantly increased when they were complexed with PS. The two ligands also had differential specificity for the TAM kinases. GAS6 preferentially bound and activated AXL in the absence of PS, but activated all 3 TAM kinases similarly in the presence of PS. In contrast, PROS1 activated TYRO3 and MERTK, but not AXL, in the presence of PS and preferentially activated TYRO3 in the absence of PS. Recent studies suggested that PROS1 may also activate AXL under some conditions [85]. PROS1 is produced by endothelial cells, hepatocytes, osteoblasts and megakaryocytes and is found in two forms in the body, bound to C4 binding protein (C4BP) or free circulating [86]. Only the free form is active and can mediate TYRO3 activation. GAS6 is produced in the kidneys, intestines, ovaries, testis, brain, bone marrow, heart, lungs and vascular endothelium [87]. Both ligands are present in plasma, although GAS6 is at much lower levels (20–50 nM) compared to other Vitamin K dependent proteins such as PROS1 (~345 nM) [88]. More recently, tubby (TUB), tubby-like protein 1 (TULP1) [89] and galectin-3 (LGALS3) [90] were identified as potential ligands for MERTK, although further studies are needed to clarify roles for these proteins in activation of MERTK in physiologic conditions and/or in disease states. Further studies are also needed to determine whether these proteins can stimulate TYRO3. Of note, TULP1 and the related protein TULP2, but not TUB, co-immunoprecipitated from cell lysates with a chimeric TYRO3 protein, consistent with roles for TULP1 and TULP2 as TYRO3 ligands [89].

### 3.2. TYRO3 Activation

Ligand binding to TYRO3 induces receptor dimerization, cross-phosphorylation of the intracellular kinase domain and increased kinase activity [39]. Structural studies revealed that TYRO3 can also homodimerize in the absence of ligand, suggesting that high levels of TYRO3 expression could be sufficient for kinase activation in some contexts [5]. In addition, TYRO3 and AXL co-immunoprecipitated from cell lysates derived from Rat2 cells with exogenous TYRO3 expression [9] and from a gonadotropin-releasing hormone (GnRH) expressing neuronal cell line, and human glioblastoma and colorectal cancer cell lines expressing endogenous TYRO3 and AXL [17,72,91]. Interaction of the endogenous proteins was enhanced following stimulation with GAS6, suggesting ligand-dependent heterodimerization [91]. Phosphorylation of AXL in response to GAS6 stimulation was also increased in Rat2 cells overexpressing TYRO3 compared to the parental cell line and this effect was abrogated when a kinase-inactive mutant of TYRO3 was overexpressed [9]. Similarly, phosphorylation of kinase-dead TYRO3 was increased in Rat2 cells expressing exogenous AXL. These data suggest that TYRO3 and AXL may cross-phosphorylate each other following heterodimerization.

TYRO3 is not only abnormally expressed in cancer cells, it is also activated. Phosphorylation of TYRO3 has been demonstrated in melanoma [49,51,52], breast cancer [37], and leiomyosarcoma [61] cell lines and in chronic lymphocytic leukemia [69] and schwannoma [55] patient samples. In breast cancer cells, high levels of phosphorylated TYRO3 correlated with sensitivity to siRNA-mediated TYRO3 inhibition [37]. In addition, stimulation with EGF ligand was required for transformation in NIH3T3 cells expressing a chimeric EGFR-TYRO3 protein [92] and transformation of RatB1a fibroblasts in response to ectopic TYRO3 expression was abrogated by a kinase-inactivating mutation [39], demonstrating an essential role for TYRO3 kinase activity in oncogenesis.

### 3.3. Downstream Signal Transduction Pathways

**Figure 2:**
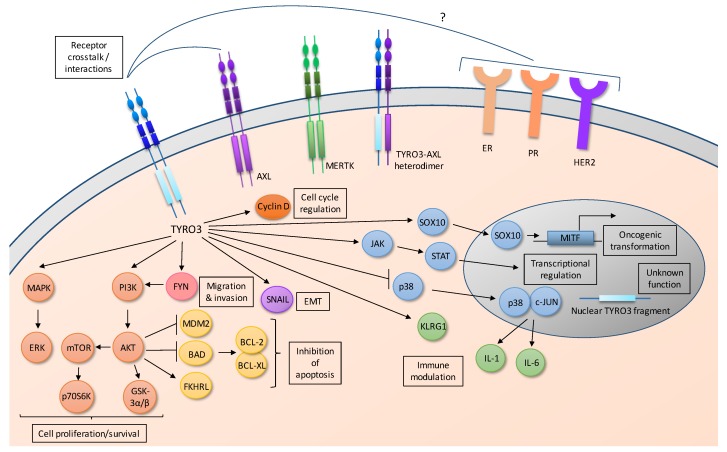
TYRO3 Cell Signaling. TYRO3 regulates pathways important for cell proliferation and survival, (e.g., PI3K/AKT/mTOR and MAPK/ERK signaling), migration and invasion, epithelial-to-mesenchymal transition (EMT), cell cycle regulation, transcriptional regulation, immune modulation and oncogenic transformation. TYRO3 and AXL heterodimerization has been reported, although their functional significance is unknown, and crosstalk between TYRO3 and estrogen receptors (ER), progesterone receptors (PR) and/or HER2 receptors has been implicated in breast cancer cells. The function of nuclear TYRO3 is unknown.

While many pathways have been well-characterized downstream of MERTK and AXL and numerous studies have described signaling changes following stimulation with TAM ligands, few studies have directly assessed signaling pathways downstream of TYRO3 [2]. Those downstream pathways that have been described play important roles in cell survival and proliferation (Figure 2). Current data suggest that, like other TAM kinases, TYRO3 functions in tumor cells to activate common oncogenic signaling pathways, particularly MEK/ERK and PI3K/AKT; however, other downstream pathways are likely to play critical roles in tumorigenesis as well. Regulation of cell-type specific effectors downstream of TYRO3 has also been reported in specific tumor types, including Microphthalmia-associated transcription factor (MITF) in melanoma 48] and alpha-fetoprotein (AFP) in hepatocellular carcinoma [45].

#### 3.3.1. TYRO3 Activates the PI3K/AKT Pathway

Numerous studies have identified the phosphatidylinositol 3 kinase (PI3K) pathway downstream of TYRO3 and identified critical roles for PI3K and its downstream effector protein kinase B (AKT) in protection from cell death. Activation of PI3K in response to stimulation with GAS6 was abrogated in platelets from *Tyro3−/−* mice and downstream signaling through AKT was also attenuated [26]. Of note, AXL phosphorylation was also abrogated in platelets from *Tyro3−/−* mice treated with GAS6 and thus, the impact of *Tyro3* loss on PI3K/AKT signaling in this context may be direct and/or indirect. However, overexpression of TYRO3 in fibroblast-like Rat2 cells was sufficient to increase phosphorylation of AKT and its downstream signaling effectors mammalian target of rapamycin (mTOR) and ribosomal protein S6 kinase beta-1 (p70S6K), suggesting a direct effect [9]. In addition, the p85 subunit of PI3K interacted with the cytoplasmic domain of TYRO3 in a yeast 2-hybrid system and p85 co-immunoprecipitated from NIH3T3 cells with a chimeric epidermal growth factor receptor (EGFR)-TYRO3 [92]. The interaction between TYRO3 and PI3K was ligand-dependent and coincided with an increase in downstream AKT phosphorylation. TYRO3-dependent phosphorylation of AKT was abrogated in the presence of the PI3K inhibitor wortmannin, indicating activation of AKT through PI3K. Downstream AKT phosphorylation was increased in NIH3T3 cells with ectopic TYRO3 expression following treatment with PROS1. Similarly, AKT phosphorylation correlated with TYRO3 expression in rat hippocampal neurons [19] and was induced in primary cortical neurons treated with PROS1, leading to downstream activation of glycogen synthase kinase 3 alpha/beta (GSK-3α/β) [15,16]. These changes were abrogated in primary neurons from *Tyro3−/−* mice [15] or in the presence of a PI3K inhibitor, LY294002 [15,16], leading to decreased protection from NMDA-induced cell death. AKT-dependent phosphorylation of pro-apoptotic forkhead transcription factor-like 1 (FKHRL1) in response to PROS1 was also abrogated in *Tyro3−/−* cortical neurons and FKHRL1 inhibition led to increased expression of first apoptosis signal (FAS) ligand, a critical activator of the extrinsic cell death pathway, and decreased protection from NMDA toxicity [15]. AKT-dependent phosphorylation of Bcl-2-associated death promoter (BAD) and mouse double minute 2 homolog (MDM2), components of the intrinsic apoptotic pathway, has also been reported in neurons in response to treatment with PROS1, suggesting regulation downstream of TYRO3; however, contributions of MERTK and AXL were not evaluated [15,16]. In brain endothelial cells, TYRO3 also mediated activation of AKT after hypoxic injury and phosphorylation of the sphingosine-1-phosphate receptor (S1P1), which associates with TYRO3 after hypoxic injury, was dependent on PI3K/AKT activation, implicating PI3K/AKT as a critical effector of TYRO3 in this context [14]. Similarly, induction of AKT phosphorylation in the sciatic nerve during myelination was abrogated in *Tyro3−/−* mice [20]. Together these data indicate regulation of the PI3K/AKT signaling pathway downstream of TYRO3, leading to AKT-dependent inhibition of both extrinsic and intrinsic apoptotic pathways and suggesting that activation of PI3K is mediated via a direct interaction between TYRO3 and p85 PI3K.

PI3K/AKT signaling has also been implicated downstream of TYRO3 in tumor cells. Phosphorylation of PI3K, AKT, and/or mTOR was decreased in hepatocellular carcinoma [46], colorectal cancer [72], and/or breast cancer [37] cell lines in response to siRNA/shRNA-mediated TYRO3 inhibition and phosphorylation of AKT following GAS6 stimulation was abrogated in melanoma cell lines treated with anti-TYRO3 antibodies. Ectopic expression of TYRO3 in the MCF10A breast cancer cell line induced AKT phosphorylation in the presence of GAS6 [38] and treatment with GAS6 promoted AKT phosphorylation in the MDA-MB435 melanoma cell line, which expresses TYRO3 but not MERTK or AXL [49]. Upregulation of TYRO3 in colorectal and sorafenib-resistant hepatocellular cancer cell lines was also associated with increased AKT phosphorylation [46,72]. Similarly, increased levels of reactive oxygen species induced both TYRO3 expression and AKT phosphorylation in taxol-resistant ovarian cancer cells [57]. In addition, AKT phosphorylation was not effectively inhibited following treatment with lapatinib in HER2-positive breast cancer cells overexpressing TYRO3 [40] and coincident inhibition of TYRO3 expression and AKT phosphorylation was observed in ovarian cancer cells following treatment with N-acetyl cysteine [57]. Moreover, treatment with a PI3K inhibitor abrogated transformation in NIH3T3 cells expressing a chimeric EGFR-TYRO3 protein and reversed TYRO3-dependent chemoresistance in MCF10A breast cancer cells, implicating PI3K as a critical downstream effector of TYRO3 oncogenic activity [38,92].

#### 3.3.2. TYRO3 Activates the SRC-Family Kinase FYN

TYRO3 activates FYN, a member of the SRC family of non-receptor tyrosine kinases that has typically been associated with signaling in T cells and neurons. A 60 kDa phosphoprotein co-immunoprecipitated with exogenously expressed TYRO3 and was recognized by an antibody with specificity for the SRC-family kinases SRC, FYN and YES [92]. Later studies using mass spectrometry demonstrated two 60 kDa proteins, FYN and killer cell lectin-like receptor subfamily G member 1 (KLRG1), that co-immunoprecipitated with the intracellular domain of TYRO3 when it was ectopically expressed in a rat Schwann cell line [20]. FYN activity was decreased in sciatic nerves from *Tyro3−/−* mice, as indicated by reduced phosphorylation on the activating Y420 residue and increased phosphorylation on inhibitory Y531, and induction of AKT phosphorylation during myelination was reduced in *Tyro3−/−* and *Fyn−/−* mice. Expression of octamer-binding factor 6 (OCT6) and early growth response 2 (EGR2/KROX20), transcription factors that are regulated downstream of AKT and play essential roles in Schwann cell myelination, was also impacted by loss of *Tyro3* or *Fyn*. Specifically, basal levels of OCT6 and induction of EGR2 were both reduced in sciatic nerves from *Tyro3−/−* and *Fyn−/−* mice. These data implicate FYN as a mediator of AKT phosphorylation downstream of TYRO3. Interestingly, like TYRO3, FYN co-immunoprecipitates with PI3K and thus, it is possible that the 3 proteins form a complex to activate downstream AKT signaling [92,93,94,95].

#### 3.3.3. TYRO3 Activates ERK1/2

The mitogen activated protein kinases extracellular signal-regulated 1 and 2 (ERK1/2) have been well-described as critical targets downstream of MERTK and AXL [96] and more limited data indicate regulation of ERK1/2 downstream of TYRO3 as well. Overexpression of TYRO3 was sufficient to induce ERK1/2 phosphorylation in fibroblast-like Rat2 cells [9]. ERK1 phosphorylation was also significantly reduced in oligodendrocytes derived from *Tyro3−/−* mice compared to wild-type oligodendrocytes, both in the presence and absence of GAS6 stimulation [18]. Similarly, siRNA-mediated TYRO3 inhibition reduced ERK1/2 phosphorylation in 3 of 6 breast cancer cell lines tested and in 2 of the 3 sensitive lines, ERK1/2 phosphorylation was accompanied by decreased levels of cyclin D1 [37], a cell cycle regulatory protein that is induced downstream of ERK1/2 in numerous cell types [97,98,99,100]. Inhibition of TYRO3 in the HEP3B hepatocellular carcinoma cell line using siRNA also reduced phosphorylation of ERK1/2 and decreased cyclin D1 levels [45]. In addition, the cytoplasmic domain of TYRO3 interacted with ran binding protein in microtubule organizing centre (RANBP9) in a yeast two-hybrid screen [101]. Cytoplasmic RANBP9 localizes to membranes where it interacts with a number of different proteins, including tyrosine kinase receptors, and interacts with the small GTPase RAN to activate RAS/MAPK signaling [102].

#### 3.3.4. TYRO3 Activates the JAK-STAT Pathway

While MERTK and AXL have established roles in JAK-STAT signaling [2], less is known about TYRO3. Inhibition of TYRO3 using siRNA decreased STAT3 phosphorylation in a HER2+ breast cancer cell line [37], suggesting stimulation of the JAK-STAT pathway in at least some cases. However, phosphorylated STAT3 was not detected in 5 additional cell lines and thus, the significance of STAT3 inhibition in this context is not clear. It also remains to be elucidated if TYRO3 activates the JAK-STAT pathway in normal physiologic conditions.

#### 3.3.5. TYRO3 Regulates Expression of MITF

Microphthalmia-associated transcription factor (MITF) plays roles in lineage-specific pathway regulation in a variety of cell types and controls expression of genes that are important for development, function, and survival in melanocytes [103]. TYRO3 was identified as a positive regulator of MITF expression in murine melanoma cells and was upregulated in 20 of 40 melanoma cell lines tested relative to other types of human cancer cell lines [48]. High levels of TYRO3 also correlated with increased MITF in this context. Ectopic expression of TYRO3 in the murine B16:F10 melanoma cell line was sufficient to increase MITF protein levels and resulted in increased nuclear localization of SOX10, a known regulator of MITF in melanoma. Induction of SOX10 nuclear localization and MITF expression was dependent on TYRO3 kinase activity and siRNA-mediated inhibition of SOX10 prevented MITF induction in response to ectopic TYRO3 expression. These observations were confirmed in human melanoma cell lines, where shRNA-mediated inhibition of TYRO3 also decreased nuclear localization of SOX10. Together these data demonstrate activation of SOX10-dependent MITF expression downstream of TYRO3.

### 3.4. Nuclear TYRO3

In addition to their traditional roles in cell signaling, numerous receptor kinases have been observed in the nucleus and non-canonical functions, such as transcriptional activation, have been described [104]. Several studies also reported nuclear localization of TYRO3. TYRO3 was present in the nucleus in approximately 40% of leiomyosarcoma patient samples that expressed TYRO3, but was not observed in tumor samples from patients with other sarcomas, including dedifferentiated liposarcomas, undifferentiated pleiomorphic sarcomas, and synovial sarcomas [61,62]. A fragment of TYRO3 that lacked the N-terminus was also detected in the nucleus in colon cancer cells and mutations in a putative nuclear localization signal (NLS) inhibited nuclear translocation [105]. Moreover, disruption of the NLS altered cellular morphology and induced apoptosis and expression of nuclear-localized TYRO3 was a poor prognostic marker in colon cancer. These data suggest kinase-independent roles for nuclear TYRO3 in tumorigenesis.

## 4. Physiologic Functions

In many cases, the normal physiologic functions for a given oncogenic protein are co-opted by cancer cells to promote tumorigenesis and TYRO3 is not an exception. The functions of TYRO3 that mediate migration of neurons to the hypothalamus during normal development and changes in platelet shape and adhesion during clot formation likely also promote tumor cell invasion, migration and metastasis. Similarly, the TYRO3 functions that promote cell survival in neurons during cell stress may promote survival of tumor cells under the conditions of nutrient deprivation and hypoxia that are hurdles to tumor growth. In addition, an understanding of the physiologic functions of TYRO3 may help to predict potential side effects of therapeutic TYRO3 inhibition. Thus, a brief review of the known functions for TYRO3 in normal physiology is provided here, with an emphasis on the phenotypes observed in *Tyro3−/−* mice. Additional roles for TYRO3 are likely to emerge as research in this area advances.

### 4.1. TYRO3 in Thrombosis

TYRO3 is expressed on the surface of platelets where it promotes aggregation and degranulation [25,26,28] and is important for clot stabilization [27]. In *Tyro3−/−* mice, thrombus size was reduced by 90% compared to wild-type mice after ligation and injection of tissue thromboplastin in the inferior vena cava [26,27] and only 25% of *Tyro3−/−* mice had a fatal thromboembolism in response to treatment with collagen and epinephrine compared to 80% of wild-type [26]. On a molecular level, TYRO3 has been implicated in inside-out signaling via integrin α(IIb)β(3), which is important for platelets to adhere to and spread on fibrinogen after initial activation [26,27]. Of note, TYRO3 knockout mice have normal bleeding time but demonstrate a tendency to re-bleed after temporary hemostasis [26].

### 4.2. TYRO3 in the Nervous System

Various roles for TYRO3 have been described in the central and peripheral nervous systems, where it is most prominently expressed under normal physiologic conditions and is present in a spectrum of cell types, including neuronal cells, oligodendrocytes, and brain epithelial cells. First, TYRO3 promotes survival of neurons and brain endothelial cells exposed to a variety of stresses such as hypoxia, serum starvation, or overstimulation with neurotransmitters [14,16,17,19,106] and loss of TYRO3 leads to increased cell death in the nervous system and neurologic pathologies [107]. For instance, a cytosine-to-thymine (C19T) mutation in the *Tyro3* gene in the *anx/anx* mouse model results in degeneration of hypothalamic neurons that normally produce appetite-stimulating neuropeptide Y (NpY), leading to anorexia [108]. Similarly, stimulation of TYRO3 protected human brain endothelial cells from hypoxic injury and prevented post-ischemic disruption of the blood brain barrier in a murine stroke model [14]. TYRO3 also plays a critical role in formation of the myelin sheath, a membrane composed of cholesterol, lipids and proteins that wraps around neuronal axons and functions as an electrical insulator [18,20]. Demyelinating diseases such as multiple sclerosis, are characterized by loss of the myelin sheath and damage to glial cells, which also protect neuronal axons [109]. In *Tyro3−/−* mice expression of myelin basic protein is reduced in oligodendrocytes and myelination is delayed and myelin thickness is reduced in both central and peripheral nerves [18,20]. Finally, TYRO3 promotes migration of neurons toward GAS6 ligand and defects in migration likely contribute to decreased numbers of GnRH neurons in the hypothalamus and resulting defects in ovulation and fertility in *Tyro3−/−* mice [17,110].

### 4.3. TYRO3 in Osteoclastic Bone Resorption

Osteoclasts are large, multinucleate cells derived from macrophages that are responsible for the bone resorption and remodeling that is critical for bone maintenance and repair and also plays a role in pathologic processes characterized by osteopenia [111]. TYRO3 is expressed in mature osteoclasts [29,30] and osteoclast numbers are reduced in *Tyro3−/−* mice, leading to a significant increase in bone mass compared to wild-type mice [112]. Similarly, bone loss was reduced in arthritic *Tyro3−/−* mice.

### 4.4. TYRO3 in the Immune System

In the immune system, the TAM receptors have critical roles in efferocytosis, the process by which apoptotic cells are engulfed and cleared by macrophages and dendritic cells. Although MERTK plays a more prominent role in macrophage-mediated efferocytosis relative to the other TAM kinases, overexpression of TYRO3 in mammary epithelial cells enhanced clearance of apoptotic cells by 2-fold compared to cells with empty vector [38] and primary peritoneal macrophages isolated from *Tyro3−/−* mice had decreased ability to phagocytose apoptotic cells compared to macrophages from wild-type mice [24]. In contrast, efferocytosis was almost completely abolished in cultures of dendritic cells (DCs) from *Tyro3−/−* mice, while loss of *Mertk* had no significant impact.

TAM kinases also function to limit the immune response. TAM kinase activation following receptor stimulation during efferocytosis promotes a type 2 immune response characterized by decreased expression of pro-inflammatory cytokines and increased expression of anti-inflammatory cytokines, leading to downregulation of inflammation and adaptive immunity to limit tissue damage and promote wound healing after clearance of pathogens [113,114]. In the absence of this self-limiting response, the immune system is hyper-activated. Indeed, *Tyro3−/−* mice have a more robust immune response and more efficient clearance of parasitic infections compared to wild-type mice [21]. However, over time persistent inflammation and immune activation lead to development of autoimmune phenotypes reminiscent of autoimmune diseases in humans, such as rheumatoid arthritis and systemic lupus erythematosus [22,24]. Of note, auto-antibodies against phospholipids have been implicated in autoimmune syndromes [22] and development of autoimmunity in TAM-knockout mice is likely also impacted by accumulation of apoptotic cells and cell debris, leading to increased levels of inflammation [115].

## 5. Functions in Cancer

### 5.1. TYRO3 in Tumorigenesis

A role for TYRO3 in malignancy has been proposed since its discovery. The human *TYRO3* gene was independently cloned from several different types of tumors, including primary teratocarcinoma cells [116], a hepatoma cell line [117], and the K562 chronic myelogenous leukemia cell line [118]. A chimeric protein consisting of the extracellular domain from EGFR and the intracellular domain of TYRO3 transformed NIH3T3 cells following stimulation with EGF ligand as indicated by anchorage-independent growth in soft agar [92]. Similarly, forced expression of TYRO3 was sufficient to confer anchorage-independent growth in RatB1a fibroblasts [39], Rat-2 fibroblasts [10], and NIH3T3 cells [35]. Moreover, RatB1a and NIH3T3 cells expressing TYRO3 formed tumors in immune-compromised mice [35,39]. Finally, overexpression of TYRO3 enabled melanoma cells to overcome senescence downstream of oncogenic BRAF(V600E) signaling [48]. These data establish TYRO3 as an oncogenic protein.

Subsequent studies to determine the impact of TYRO3 inhibition in a variety of tumor types support roles for TYRO3 in tumor cell proliferation and survival (Table 1). Inhibition of TYRO3 using siRNA or shRNA decreased expansion of colorectal cancer [36,72], hepatocellular carcinoma [45,46], melanoma [48,49], breast cancer [37], ovarian cancer [57], leiomyosarcoma [61], and AML [68] cell lines in culture and forced expression of TYRO3 was sufficient to increase cell density in cultures of the MCF10A breast cancer cell line [38]. Similarly, in a large-scale screen to identify tyrosine kinases with potential for therapeutic targeting in a panel of 484 primary AML patient samples, 7.6% of samples exhibited reduced cell density in culture following siRNA-mediated TYRO3 inhibition and TYRO3 was identified as one of the top two hits [68]. Interestingly, the role for TYRO3 in breast cancer appears to be limited to hormone receptor positive (HR+) tumors, as siRNA-mediated TYRO3 inhibition reduced cell density in cultures of estrogen receptor or HER2 positive cell lines, both in the presence and absence of hormone stimulation, but had no significant impact on expansion of triple negative cell lines [37]. These data suggest the possibility of crosstalk between TYRO3 and hormone receptors in HR+ breast cancers (Figure 2).

Changes in tumor cell density in response to TYRO3 inhibition are in part due to inhibition of tumor cell proliferation. BrdU incorporation was reduced in thyroid cancer cell lines with siRNA-mediated TYRO3 inhibition [47]. Similarly, HR+ breast cancer cell lines expressing siTYRO3 exhibited an increased fraction of cells in G0/G1 phase of the cell cycle and concurrent decreases in the numbers of cells in S and G2/M phases [37]. These changes were associated with decreased expression of the cell cycle regulatory protein Cyclin D1, which promotes progression through G1/S and among the 3 D-type cyclin family members has been most predominantly associated with tumorigenesis [119]. Cyclin D1 levels were also decreased in hepatocellular carcinoma cells expressing TYRO3 siRNA [45]. Similarly, treatment with crizotinib or foretinib, small molecule tyrosine kinase inhibitors with activity against TAM kinases, resulted in decreased cell density in leiomyosarcoma cell line cultures and this was accompanied by changes in cell cycle progression leading to accumulation of cells in G1 or G2 phases [61]. In several cell lines a population of cells with >4N DNA content was also observed, suggesting induction of polyploidy. While both crizotinib and foretinib target numerous other kinases, including MERTK and AXL, the degree of impact of these agents correlated with the degree of TYRO3 inhibition, suggesting TYRO3 as a target in this context.

Inhibition of TYRO3 also promoted apoptosis. Derivatives of the A2058 and SKMEL-2 melanoma cell lines expressing shTYRO3 exhibited increased apoptosis in culture relative to cells expressing a control shRNA [48,49]. Similarly, thyroid cancer cell lines expressing siTYRO3 exhibited increased apoptosis in response to serum starvation [47]. Treatment with a neutralizing TYRO3 antibody was sufficient to induce apoptosis in cultures of the HCT-116 colorectal cancer line and colorectal cancer patient samples [35]. Treatment with the TAM kinase inhibitors crizotinib or foretinib also induced apoptosis in 5 of 6 leiomyosarcoma cell lines tested [61].

Similar data demonstrate critical roles for TYRO3 in oncogenic phenotypes and tumorigenesis (Table 1). Inhibition of TYRO3 using shRNA in the HCT116 colorectal cancer [35], A2058 melanoma [48], SK-LMS-1 leiomyosarcoma [61], and paclitaxel-resistant SKOV3/TR ovarian cancer [57] cell lines resulted in decreased colony-formation in soft agar demonstrating a role for TYRO3 in anchorage-independent growth, which is a classic feature of cellular transformation. Moreover, tumor volume and weight were significantly reduced in immune-compromised mice with a subcutaneous xenograft of HCT116 cells expressing shTYRO3 compared to mice injected with HCT116 cells expressing a control shRNA and in mice with subcutaneous HCT-116 xenografts treated with an anti-TYRO3 antibody compared to mice treated with a control IgG antibody [35]. In addition, tumors formed in only 8 of 12 mice injected with cells expressing shTYRO3 while tumors formed in all mice injected with control cells. Similarly, tumor formation was significantly delayed in immune-compromised mice with intradermal xenografts of the A2058 melanoma cell line expressing shTYRO3 and only 3 of 10 mice injected with shTYRO3 cells formed detectable tumors while the control cell line formed tumors in all 5 mice tested [48]. These data implicate TYRO3 as a therapeutic target in cancer.

### 5.2. TYRO3 in Metastasis

Several studies have implicated roles for TYRO3 in oncogenic phenotypes related to metastasis (Table 1). In order to escape the primary tumor site, cancer cells must undergo a phenotypic shift called epithelial-mesenchymal transition (EMT), whereby epithelial cells lose their polarity and cell-cell adhesions and assume a mesenchymal phenotype associated with increased migratory and invasive properties [120]. This shift in phenotype allows tumor cells to migrate out of the primary tumor and into the circulation, such that they can travel to distant sites where they may establish and proliferate to generate a metastatic tumor.

Inhibition of TYRO3 in hepatocellular and colorectal carcinoma cell lines using siRNA decreased both migration and invasion in trans-well and/or scratch-wound assays [46]. Conversely, inhibition of miR-7 microRNA, an upstream regulator of TYRO3 in this context, resulted in increased TYRO3 expression and increased migration and invasion and these effects were abrogated in the presence of siTYRO3. Similarly, shRNA-mediated TYRO3 inhibition in the HCT-116 and HT-29 colorectal cancer cell lines decreased migration and invasion and overexpression of TYRO3 in HT-29 cells was sufficient to enhance migration [35]. Overexpression of TYRO3 was also sufficient to induce transcriptional changes characteristic of EMT, including decreased expression of the epithelial markers β-catenin, E-cadherin and ZO-1 and increased expression of mesenchymal proteins N-cadherin and α-smooth muscle actin. In addition, overexpression of TYRO3 induced expression of SNAI1, a transcriptional repressor that plays a central role in EMT [121], and shRNA-mediated inhibition of TYRO3 decreased SNAI1 expression. Moreover, shRNA-mediated inhibition of SNAI1 abrogated EMT transcriptional changes and migratory and invasive phenotypes associated with TYRO3 overexpression, indicating SNAI1 as a mediator of these effects. Consistent with these data, in a meta-analysis of microarray data from 688 colorectal tumors, TYRO3 expression was positively correlated with mesenchymal markers SNAI1, N-cadherin, and fibronectin and negatively correlated with epithelial markers E-cadherin and Occludin. Similarly, TYRO3 was upregulated 1.5 to 2.25-fold in non-small cell lung cancer cells stimulated with EMT inducers TGFβ, SNAI1 or ZEB [42]. GAS6 ligand was also upregulated indicating autocrine or paracrine stimulation of TYRO3. Together these data suggest that TYRO3 functions in tumor cells to promote metastasis. Indeed, the incidence of metastatic lesions in the lung was significantly reduced in immune-compromised mice with a subcutaneous xenograft of HCT116 cells expressing shTYRO3 compared to mice injected with HCT116 cells expressing a control shRNA [35]. Interestingly, TYRO3 expression was increased in a bone metastatic lesion compared to dormant bone marrow tumor cells isolated from a non-metastatic limb from the same mouse when Du145 prostate cancer cells were implanted subcutaneously on a collagen scaffold and the primary tumor was resected to allow de novo development of metastatic lesions [60]. In contrast, TYRO3 expression was not increased when the tumor cells were implanted by cardiac injection, suggesting selection for high TYRO3 expression during the initial steps of metastasis in this model and consistent with the demonstrated roles for TYRO3 in EMT.

### 5.3. TYRO3 in Therapeutic Resistance

Consistent with the known roles for TYRO3 in tumor cell survival, TYRO3 has been implicated as a mediator of resistance to both traditional cytotoxic chemotherapies and molecularly-targeted agents (Table 1). Inhibition of TYRO3 increased sensitivity to cytotoxic chemotherapies in a variety of cancer cell lines in cell culture models. Derivatives of the A2058 melanoma cell line with siRNA-mediated TYRO3 inhibition exhibited increased apoptosis relative to cells expressing a control siRNA in response to treatment with cisplatin, temozolomide, or docetaxel [48]. Similarly, HCT-116 colorectal cancer cells expressing siTYRO3 were more sensitive to treatment with 5-fluorouracil [35,36] and induction of apoptosis in response to 5-fluorouracil, paclitaxel, or oxaliplatin was significantly increased in cultures treated with an anti-TYRO3 antibody [35]. Treatment with 5-fluorouracil, paclitaxel, or oxaliplatin in combination with anti-TYRO3 antibody also induced apoptosis in cultures of colorectal patient samples more effectively than either single agent. Moreover, treatment with anti-TYRO3 antibody enhanced sensitivity to 5-fluorouracil in vivo in mice with a subcutaneous HCT-116 colon cancer xenograft. Thus, TYRO3 mediates intrinsic resistance to cytotoxic chemotherapies. TYRO3 has also been implicated in acquired resistance to chemotherapy. Overexpression of TYRO3 in the MCF10A breast cancer cell line decreased sensitivity to camptothecin [38]. TYRO3 was also upregulated in paclitaxel-resistant and cisplatin-resistant derivatives of the SKOV3 and A2780 ovarian cancer cell lines, respectively, relative to parental cell lines and siRNA-mediated inhibition of TYRO3 reduced tumor cell expansion in cultures of the paclitaxel-resistant SKOV3 cell line [56,57]. In contrast, MERTK and AXL levels were decreased. In cultures of the paclitaxel-resistant SKOV3 cell line, therapeutic effects mediated by N-acetylcysteine (an antioxidant that neutralizes reactive oxygen species), apigenin (a dietary flavone), and metformin (an FDA-approved agent used for treatment of diabetes) correlated with reduced TYRO3 expression [56,57,58]. In addition, there was a trend toward increased metformin-sensitivity in cells with siRNA-mediated inhibition of TYRO3, although statistical significance was not reported [56].

TYRO3 can also mediate acquired resistance to molecularly-targeted therapies. A derivative of the Huh-7 hepatocellular carcinoma cell line with acquired resistance to the multi-kinase inhibitor sorafenib had increased expression of TYRO3 relative to the parental cell line [46]. Sorafenib-resistant cells also exhibited increased migration and invasion and induction of an EMT transcriptional program. Inhibition of TYRO3 in sorafenib-resistant Huh-7 cells using siRNA decreased tumor cell expansion relative to cells expressing a control siRNA and treatment with a combination of sorafenib and siTYRO3 was significantly more effective than either treatment alone. In addition, exogenous expression of miR-7, which mediates changes in migration and invasion via downregulation of TYRO3 in parental Huh-7 cells, was sufficient to reduce migration and invasion in sorafenib-resistant Huh-7 cells and completely restored sensitivity to sorafenib. These data suggest upregulation of TYRO3 as a mechanism of resistance to sorafenib in this cell line. TYRO3 has also been implicated as a mediator of resistance to HER2 inhibitors in breast cancer. High levels of TYRO3 expression correlated with poor response to therapy in patients with HER2-positive breast cancer and exogenous expression of TYRO3 was sufficient to confer resistance to the small molecule HER2 inhibitor lapatinib in 2 of 3 breast cancer cell lines [40]. TYRO3 was also upregulated in a lapatinib-resistant derivative breast cancer cell line relative to the parental line and in an intrinsically-resistant breast cancer cell line treated with the HER2-targeted antibody trastuzumab. Moreover, TYRO3 was upregulated in paired post-treatment biopsies from patients with trastuzumab-resistant tumors, but was not upregulated in post-treatment biopsies from trastuzumab-sensitive tumors.

### 5.4. TYRO3 in the Tumor Microenvironment

Tumorigenesis is influenced by the complex interplay of signals between tumor cells and the tumor microenvironment. As described above, TAM kinase activation in macrophages following receptor stimulation during efferocytosis promotes an anti-inflammatory type 2 immune response that facilitates wound healing. In the context of cancer, efferocytosis in the tumor microenvironment similarly leads to induction of anti-inflammatory type 2 cytokines and this shift in phenotype in the tumor microenvironment can suppress anti-tumor immunity [122]. At the same time, stimulation of TAM receptors on tumor cells promotes their proliferation, survival, migration, invasion, and other oncogenic phenotypes. Thus, combined activation of TAM kinase signaling in immune cells and cancer cells can promote tumor growth and progression.

Cross talk between tumor cells and tumor-associated macrophages can educate macrophages to express TAM family ligands [123]. For instance, in a syngeneic murine colon cancer model, circulating leukocytes and tissue-resident macrophages expressed minimal GAS6, but GAS6 was dramatically upregulated in tumor-associated macrophages [124]. In addition, tumor growth was significantly inhibited in *Gas6−/−* mice, demonstrating a pro-tumorigenic role for GAS6 in the tumor microenvironment. Stimulation of murine macrophages with IL-10 or M-CSF, cytokines that are expressed by type 2 macrophages, was sufficient to induce GAS6 expression. GAS6 expression was also upregulated in murine macrophages in response to treatment with the chemotherapeutic agent 5-fluorouracil and co-culture with macrophages or treatment with GAS6 reduced the impact of treatment with 5-fluorouracil on human colon cancer cells, revealing a chemo-protective role for GAS6 produced by macrophages in the tumor microenvironment [36].

Interactions between tumor cells and the tumor microenvironment can also educate tumor cells to express TAM family ligands. Treatment with IFN-γ, which induces a pro-inflammatory type 1 (M1) phenotype in macrophages, also induced expression of PROS1 in 6 of 8 murine melanoma, lung cancer, breast cancer, and pancreatic cell lines tested and treatment with PROS1 or co-culture with wild-type B16.F10 melanoma cells, but not B16.F10 cells with CRISPR-mediated PROS1 knock-down, decreased M1 cytokine expression in M1-polarized macrophages [125]. Moreover, decreases in M1 cytokine expression were not observed in macrophages from *Tyro3−/−* or *Mertk−/−* mice co-cultured with B16.F10 melanoma cells, implicating critical immunosuppressive roles for both TYRO3 and MERTK in the tumor microenvironment. Thus, tumor cells produce PROS1 in response to pro-inflammatory M1 cytokines and PROS1 stimulates TYRO3 and MERTK on macrophages to promote an anti-inflammatory M2 phenotype, thereby repressing the innate immune response in the tumor microenvironment. In these studies, the biochemical mechanisms by which TYRO3 activation can suppress the inflammatory response in the tumor microenvironment were also assessed [125]. In macrophages, both activating and inhibitory forms of p38 MAPK- p38α and p38γ, respectively- are expressed and the two forms were inversely regulated in response to PROS1- i.e., phosphorylated p38α decreased and phosphorylated p38γ increased. The net result of PROS1 stimulation was a shift from a predominance of c-JUN transcription factor complexes with activated phospho-p38α to a predominance of c-JUN complexes with inhibitory phospho-p38γ, leading to reduced downstream transcriptional activation of inflammatory cytokines such as IL-1 and IL-6 (Figure 2). These studies reveal novel modes of TAM kinase signaling through inhibition of p38 MAPK activity to turn down expression of pro-inflammatory M1 cytokines and suppress anti-tumor immunity. Indeed, treatment with the p38 activator anisomycin abrogated the effects of PROS1 on M1 cytokine expression, implicating a critical role for p38 downstream of TYRO3.

### 5.5. Prognostic Significance of TYRO3

TYRO3 expression portends poor prognosis in several malignancies. Higher levels of TYRO3 expression correlated with worse overall survival in patients with colorectal cancer in two independent data sets [35,36] and in patients with hepatocellular carcinoma [46]. Higher levels of TYRO3 expression also correlated with poor response to therapy in patients with HER2-positive breast cancer in a meta-analysis of 22 publicly available microarray data sets [40]. In this study, patients with TYRO3 expression levels greater than the median had decreased progression-free survival. In patients with hepatocellular carcinoma, TYRO3 expression was significantly associated with higher levels of serum and intratumoral alpha-fetoprotein [45,46], which is an independent diagnostic predictor of disease stage, disease progression, and poorer overall survival [126]. TYRO3 expression also correlated with tumor size of 3 cm or higher; however, on multivariate analysis this association was not statistically significant, potentially due to the limited sample size in this study (*n* = 55) [45]. As noted above, expression of nuclear-localized TYRO3 was a poor prognostic marker in colon cancer [105].

## 6. TYRO3 as a Therapeutic Target

### 6.1. Translational Agents Targeting TYRO3

The demonstrated roles for TYRO3 in tumorigenesis, metastasis and therapeutic resistance in a variety of tumor types and emerging roles in immune suppression in the tumor microenvironment implicate TYRO3 as a therapeutic target in cancer and development of translational agents to target TYRO3 will allow direct testing of this idea. Multi-kinase inhibitors with activity against TYRO3 have been described and are in various stages of development with the intent of targeting other oncogenic kinases (Table 2) [127,128,129,130,131,132,133,134,135,136,137,138,139,140,141,142,143,144,145]. In particular, numerous agents with inhibitory activity against MET also have potent activity against TYRO3, including BMS-777607 [127], foretinib [130,132], LDC1267 [133], LY2801653 [134], RXDX-106 [138] and sitravatinib [139]. In addition, the SRC/ABL kinase inhibitor bosutinib [128,129,130] and the VEGFR2 inhibitor vandetinib [130] are both FDA-approved and have activity against TYRO3. All of these agents also target MERTK and/or AXL with similar potencies. More recently, several compounds designed to target MERTK have been described and these have activity against both TYRO3 and AXL at 10–50 fold higher concentrations [135,136,140,141,142,143,144,145].

Due to the overlapping functions shared by the TAM kinases and the progressive phenotypes observed in mice lacking multiple family members, agents targeting multiple TAM kinases may have increased side effects relative to agents that are selective for individual family members [2]. Thus, development of agents that target TYRO3 with greater selectivity, particularly relative to the other TAM kinases, is desirable. High-throughput screening identified spiroindoline-2-carboxyindoles and 2,4-diaminopyrimidine-5-carboxamides as the first TYRO3-selective tyrosine kinase inhibitors [146,147,148]. Subsequent structure-activity relationship (SAR) studies on the 2,4-diaminopyrimidine-5-carboxamide backbone led to development of compounds with enhanced selectivity [146,147]. Of these, Compound 32 had greater than 100-fold selectivity for TYRO3 in a panel of 31 tyrosine kinases, including AXL, but was not as selective against MERTK (<10-fold). In addition, Compound 32 was not very potent (IC_50_ = 70 nM) and had poor metabolic stability in liver microsome assays. Further SAR optimization led to discovery of derivative compounds with better activity and selectivity, reduced clearance in microsome assays, and improved solubility, including Compound 21, which is the most potent TYRO3 kinase inhibitor described to date (IC_50_ = 0.7 nM), and Compound 19, which has greater than 100-fold selectivity for TYRO3 (IC_50_ = 10 nM) over both MERTK and AXL [146,147]. However, these compounds have poor membrane permeability and oral bioavailability. More recently a TYRO3-selective pyrrolopyrimidine-based inhibitor, Compound 5, was described; however, additional SAR studies are needed to optimize the selectivity of this series for TYRO3 (IC_50_ = 6.7 nM) relative to MERTK (IC_50_ = 19 nM) [149]. In addition, the pharmacokinetic properties of Compound 5 are unknown. Thus, more work is needed to develop selective and potent TYRO3 inhibitors that are optimized for in vivo and clinical applications.

Neutralizing antibodies targeting TYRO3 have also been described. These include two different mouse monoclonal antibodies raised against the extracellular domain of human TYRO3 that block GAS6-induced TYRO3 signaling in melanoma cells and have low cross-reactivity with MERTK and AXL [47,49]. A humanized antibody has also been described and extensive preclinical studies demonstrated reversal of TYRO3-dependent EMT phenotypes, induction of cell death, and increased sensitivity to cytotoxic chemotherapies in colorectal cancer cell lines and/or patient samples treated with the antibody. Moreover, tumor growth was significantly reduced and sensitivity to 5-fluorouracil was significantly increased in response to treatment with the antibody in a cell line-based murine xenograft model, providing the first demonstration of in vivo therapeutic activity mediated by a translational agent that selectively targets TYRO3.

Ligand sinks containing the MERTK or AXL extracellular domain are also available, but would be expected to target other TAM family members [34]. Exogenous expression of miR-7 has been used to downregulate TYRO3, but again would be expected to impact other proteins as well [46,72].

### 6.2. Potential Side Effects of TYRO3 Inhibition

The physiologic functions mediated by TYRO3 and the phenotypes observed in *Tyro3−/−* mice suggest potential side effects of TYRO3 inhibition. In particular, *Tyro3−/−* mice exhibit decreased thrombosis, neurologic abnormalities, reduced myelination, and defects in spermatogenesis and ovarian function; however, in most cases the phenotypes observed in *Tyro3−/−* knock-out mice are mild and only become pronounced when two or three family-members are inhibited. For instance, while mice with a deficiency in any one of the three TAM-family kinases have mild defects in platelet aggregation, this effect is more pronounced when 2 of the 3 receptors are disrupted and is further exacerbated when all 3 receptors are absent [25,26,27]. Similarly, mice lacking any one of the TAM kinases develop enlarged spleens and lymph nodes and these autoimmune phenotypes are most pronounced in triple knock-out mice, where hyperactivated B and T cells fill both compartments and form colonies in essentially every major organ [22]. Survival in the absence of growth factors and migration toward GAS6 were also more significantly impaired in neurons with combined inhibition of TYRO3 and AXL compared to inhibition of TYRO3 or AXL alone [17]. Thus, the risk of side effects is expected to be significantly reduced for agents that selectively target TYRO3 relative to less selective agents that target 2 or all 3 of the TAM kinases.

Despite increased potential for side effects, in some cases it may be beneficial to target more than one of the TAM kinases. For instance, inhibition of either TYRO3 or AXL via siRNA decreased expansion of thyroid cancer cells in culture and induced apoptosis, but combined inhibition of both receptors had an even greater effect [47]. In addition, TYRO3/AXL and potentially TYRO3/MERTK heterodimers may be more effectively inhibited when both proteins are targeted.

## 7. Conclusions

TYRO3 is expressed in a wide variety of cancer types and mediates downstream signaling through PI3K/AKT, MAPK/ERK and other oncogenic pathways to promote tumor cell survival and proliferation, motility, and EMT. High levels of TYRO3 expression have been associated with poor prognosis. Inhibition of TYRO3 reduces tumor growth and metastasis in animal models. TYRO3 also mediates intrinsic and acquired resistance to chemotherapy and molecularly-targeted agents. TYRO3 is expressed on macrophages in the tumor microenvironment, where it functions to suppress anti-tumor immunity. These data implicate TYRO3 as a therapeutic target, both in tumor cells and in the tumor microenvironment, and provide rationale for development of translational agents that selectively and potently target TYRO3. Efforts to develop TYRO3-selective small molecule inhibitors and neutralizing antibodies suitable for clinical application are ongoing.

## Figures and Tables

**Table 1:** TYRO3 expression and function in cancer.

Cancer	Expression		Functional Roles								Reference(s)
	Cell Lines	Patient Samples	Cell Proliferation and/or Survival	Anchorage-Independent Colony Formation	Xenograft Model	Migration and/or Invasion	EMT	Metastasis	Drug Resistance	Unfavorable Prognosis	
**Hepatocellular carcinoma**	OX	OX	+								[45]
		OX									[33]
	+	OX	+			+			+ ^*^	+	[46]
**Colorectal cancer**	+	OX	+					+	+	+	[36]
		+	+	+	+	+	+	+	+	+	[35]
	OX		+			+					[72]
**Esophageal Cancer**	+	OX									[63]
**Melanoma**	+										[50]
	+	OX	+	+	+				+		[48]
	+										[51]
	+										[52]
	+										[53]
	+	OX	+								[49]
**Thyroid Cancer**	Ectopic		+								[47]
**Lung cancer**	+										[41]
	+	+									[44]
	+										[43]
							+				[42]
**Prostate cancer**	OX	OX									[59]
	+							+			[60]
**Breast cancer**	OX										[39]
	+		+								[37]
	+		+						+		[38]
		+							+ ^#^	+	[40]
**Ovarian Cancer**	+		+						+		[57]
	+								+		[56]
	+								+		[58]
**Endometrial Cancer**		+									[64]
**Leiomyosarcoma**		OX									[62]
	+	+	+	+							[61]
**Dedifferentiated Liposarcoma**		+									[61]
**Undifferentiated Pleomorphic Sarcoma**		+									[61]
**Synovial Sarcoma**		+									[61]
**Schwanomma**		+									[54]
	OX										[55]
**Multiple Myeloma**	+										[65]
**AML**	+	+									[66]
	+										[67]
	+		+								[68]
**ALL (B-cell & T-cell)**	Ectopic										[66]
	+										[67]
**CML**	+										[66]
**CLL**		OX									[69]

OX = Overexpressed in tumor tissue/cells relative to normal tissue/cells; * = sorafenib resistance; # = trastuzumab resistance; EMT = epithelial-mesenchymal-transition.

**Table 2:** Translational agents targeting TYRO3. Tyrosine kinase inhibitors with IC_50_ or K_d_ values less than 100 nM are shown. Unless otherwise indicated values refer to activity in enzymatic assays. (* Cell-based assay, *ND* = Not Determined).

Compound	Primary Target	TYRO3 Activity	MERTK Activity	AXL Activity	Other Targets	Development Phase	Indications	Reference(s)
BMS-777607/ASLAN002	MET	IC_50_ = 4.3 nM	IC_50_ = 14 nM	IC_50_ = 1.1 nM	RON, AURKB, FLT3	Phase I	Advanced solid tumors	[127]
Bosutinib (SKI-606, PF-5208763)	SRC, ABL	K_d_ = 61 nM	K_d_ = 110 nM	K_d_ = 52 nM	LYN, HCK, TEC, STE20K, CAMK2G	Approved	Breast cancer, glioblastoma, Ph+ CML	[128,129,130]
C52		IC_50_ = 96 nM	IC_50_ = 110 nM	IC_50_ = 140 nM				[131]
Foretinib	MET, VEGFR2	K_d_ = 2 nM	K_d_ = 0.3 nM	K_d_ = 0.1 nM	RON, PDGFRβ, KIT, FLT3, TIE2		Breast cancer, NSCLC	[130,132]
LDC1267	TYRO3, MERTK, AXL	IC_50_ = 8 nM	IC_50_ < 5 nM	IC_50_ = 29 nM	MET, AURKB, LCK	Preclinical	Metastatic melanoma	[133]
LY2801653	MET	IC_50_ = 28 nM *	IC_50_ = 0.8 nM	*ND*	MST1R, FLT3, TEK, ROS, DDR1/2	Phase I	Advanced cancer	[134]
MRX-2843	MERTK, FLT3	IC_50_ = 17 nM	IC_50_ = 1.3 nM	IC_50_ = 15 nM	TRKA, LOK	Phase I	Advanced solid tumors	[135,136]
ONO-7475 (ONO-9330547)	FLT3, TYRO3, MERTK, AXL	IC_50_ = 1.9 nM	IC_50_ = 0.4 nM	IC_50_ = 2.2 nM		Phase I	Acute leukemia	[137]
RXDX-106 (CEP-40783)	TYRO3, MERTK, AXL, MET	IC_50_ = 19 nM	IC_50_ = 29 nM	IC_50_ = 7 nM		Phase I	Advanced solid tumors	[138]
Pfizer Compound 19	TYRO3	IC_50_ = 10 nM	*ND*	*ND*			Thrombosis	[146]
Pfizer Compound 21	TYRO3	IC_50_ = 0.7 nM	*ND*	*ND*	MERTK		Thrombosis	[146]
Pfizer Compound 32	TYRO3	IC_50_ = 70 nM	*ND*	*ND*	MERTK		Thrombosis	[147]
Sitravatinib (MGCD516)	TYRO3, MERTK, AXL, VEGFR, PDGFR, KIT	IC_50_ < 1 nM	IC_50_ < 1 nM	IC_50_ < 1 nM	MET, RET	Phase I/II	Urethlial carcinoma, liposarcoma, advanced cancer, NSCLC	[139]
UNC569	MERTK	IC_50_ = 48 nM	IC_50_ = 2.9 nM	IC_50_ = 37 nM	FLT3, MAPKAPK2, RET		ALL	[140,141]
UNC1062	MERTK	IC_50_ = 60 nM	IC_50_ = 1.1 nM	IC_50_ = 85 nM	FLT3		Metastatic melanoma	[142,143]
UNC1666	MERTK, FLT3	IC_50_ = 29 nM	IC_50_ = 0.55 nM	IC_50_ = 37 nM	TRKA, TRKB, TRKC		AML	[144]
UNC2025	MERTK, FLT3	IC_50_ = 18 nM	IC_50_ = 0.7 nM	IC_50_ = 7.5 nM	TRKA, TRKC	Preclinical	ALL, AML	[136]
UNC3133	MERTK	IC_50_ = 57 nM	IC_50_ = 8 nM	IC_50_ = 22 nM	FGFR1, ITK, KDR, PDGRα, TRKA, AURKA, p70S6K			[145]
UNC Compound 5	TYRO3	IC_50_ = 6.7 nM	IC_50_ = 19 nM	IC_50_ = 206 nM	MERTK			[149]
Vandetinib	VEGFR2, VEGFR3, EGFR	K_d_ = 93 nM	K_d_ = 1400 nM	K_d_ = 250 nM	RET	Approved	Thyroid cancer, NSCLC	[130]
